# 149. Impact of Stewardship on Antibiotic Utilization Rates During the COVID-19 Pandemic: Successes and Challenges in a Regional Hospital

**DOI:** 10.1093/ofid/ofab466.351

**Published:** 2021-12-04

**Authors:** Ana Macias, Jennifer Elgin, Donna Duerson, Cirle A Warren

**Affiliations:** 1 Novant Health- University of Virginia Culpeper Medical Center, Culpeper, VA; 2 University of Virginia, Charlottesville, VA

## Abstract

**Background:**

Antibiotic stewardship (AS) is at the core of patient safety and prevention of antimicrobial resistance. Healthcare providers prescribe antibiotics for COVID-19 despite low rates of bacterial co-infection. Our regional hospital had antibiotic utilization (AU) rates higher than other health systems even prior to the emergence of SARS-Cov2. We analyzed the effect AS on AU during the pandemic.

Total Antibiotic Utilization Rates Before and During COVID-19 Pandemic

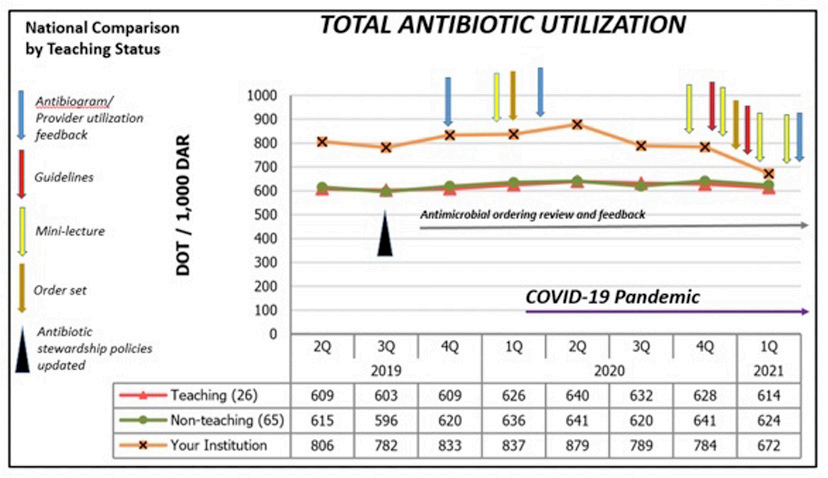

**Methods:**

Total and specific AU rates were benchmarked using BD MedMined’s medication analytics system from 2^nd^ quarter 2019 to 1^st^ quarter 2021. The AS team released yearly antibiogram and individual prescriber’s AU rates and performed weekly, and as needed, review of antibiotic ordering and feedback. To assist in appropriate prescribing decisions, remote educational sessions or mini-lectures and local antibiotic guidelines were developed during the pandemic period. AU rates were monitored quarterly to determine the effects of the AS interventions to prescribing practices.

**Results:**

Total and specific AU rates were higher (up to 34% and 80%, respectively) in our index hospital compared to other non-teaching hospitals nationally prior to the pandemic. Total antibiotic utilization increased by only 5.5% in the 2^nd^ quarter 2020, peak of AU during the pandemic. Total, vancomycin, piperacillin-tazobactam and quinolone utilization rates decreased by 19%, 41%, 38%, and 52%, respectively, at 1^st^ quarter 2021 compared to 4^th^ quarter 2019. Steeper decreases were noted with implementation of educational activities. Ceftriaxone use remained high and was 50% greater than comparator hospitals at 1^st^ quarter 2021.

**Conclusion:**

Although problematic during the COVID-19 pandemic, AS can have significant impact on provider prescribing practices and decrease total and specific antibiotic utilization rates. The use of ceftriaxone, an antibiotic commonly used for empiric bacterial coverage for community acquired pneumonia, presents as a continuing challenge.

**Disclosures:**

**All Authors**: No reported disclosures

